# Infra-red thermometry: the reliability of tympanic and temporal artery readings for predicting brain temperature after severe traumatic brain injury

**DOI:** 10.1186/cc7898

**Published:** 2009-05-27

**Authors:** Danielle Kirk, Timothy Rainey, Andy Vail, Charmaine Childs

**Affiliations:** 1 Brain Injury Research Group, School of Translational Medicine, University of Manchester, Salford Royal Foundation Trust, Stott Lane, Salford, M6 8HD UK; 2 Biostatistics Group, University of Manchester, Salford Royal Foundation Trust, Stott Lane, Salford, M6 8HD UK

## Abstract

**Introduction:**

Temperature measurement is important during routine neurocritical care especially as differences between brain and systemic temperatures have been observed. The purpose of the study was to determine if infra-red temporal artery thermometry provides a better estimate of brain temperature than tympanic membrane temperature for patients with severe traumatic brain injury.

**Methods:**

Brain parenchyma, tympanic membrane and temporal artery temperatures were recorded every 15–30 min for five hours during the first seven days after admission.

**Results:**

Twenty patients aged 17–76 years were recruited. Brain and tympanic membrane temperature differences ranged from -0.8 °C to 2.5 °C (mean 0.9 °C). Brain and temporal artery temperature differences ranged from -0.7 °C to 1.5 °C (mean 0.3 °C). Tympanic membrane temperature differed from brain temperature by an average of 0.58 °C more than temporal artery temperature measurements (95% CI 0.31 °C to 0.85 °C, *P *< 0.0001).

**Conclusions:**

At temperatures within the normal to febrile range, temporal artery temperature is closer to brain temperature than is tympanic membrane temperature.

## Introduction

Temperature measurement is important during routine neurocritical care. There is retrospective evidence that moderate to high body temperature is an independent predictor of intensive care unit (ICU) and hospital length of stay and leads to a higher mortality and worse outcome in a mixed population of neurosurgical ICU patients [1]. Recent prospective data of brain temperature and outcome in a relatively homogenous population of patients with severe traumatic brain injury (TBI) show that outcome is worse at temperature extremes (high and low) [2]. Current opinion favours treatment of pyrexia in patients with neurological injury. However, there are no published guidelines or recommendations for the management of raised temperature [3]. The focus of the most recent (2007) Brain Trauma Foundation (BTF) guidelines for the management of temperature after human TBI was on the management of hypothermia (a treatment which is limited to a level III recommendation only [4]). Popular opinion has, therefore, considered controlled normothermia as a clinical therapeutic option, but whether normothermia has the potential for therapeutic benefit for the TBI patient remains untested.

As body core temperature frequently dissociates from brain temperature [5,6] there remains some doubt about the reliability of traditional body temperature methods for brain temperature estimation. While measurement of deep body core temperature using traditional monitoring sites such as rectum, oesophagus, urinary bladder or pulmonary artery might be expected to provide a reasonable surrogate for brain temperature during neurocritical care, we have shown that tympanic membrane temperature is currently the most popular, non-surgical method of brain temperature estimation in UK neurosurgical practice [7]. Thus, most (70%) neurosurgical centres do not measure 'true' body core temperature, rather they employ a measurement method about which serious doubts regarding accuracy are documented [8-10].

A new, non-invasive method for core temperature estimation is now available which captures infra-red heat energy from the skin overlying the course of the temporal artery [11]. The technique is quick; the instrument is easy to clean and is relatively inexpensive. The aim of the study was to determine if infra-red temporal artery thermometry provides a better performance as a 'surrogate' for brain temperature than the most common method (tympanic temperature) used in UK neurocritical care.

## Materials and methods

The study was approved by the local research ethics committee (reference number 06/Q1406/115). Approval to undertake the study was obtained from the patient's spouse, relative or partner before measurements were made.

### Patients

All patients aged 16 years or above, who were admitted to our 16 bed, level 3, university teaching hospital ICU within 24 hours of severe TBI were eligible for recruitment to the study.

Patients were admitted either as direct referrals from the emergency department (ED) or as tertiary referrals from EDs of other hospitals within the greater Manchester region. All the patients were sedated, intubated and mechanically ventilated; all had an intra or extra-axial lesion on computed tomography (CT), with or without systemic trauma and a Glasgow Coma Scale (GCS) of eight or less on admission to the ICU. The patients were treated in accordance with local neurointensive care guidelines to maintain cerebral perfusion pressure at 60 mmHg or higher and intracranial pressure (ICP) below 20 mmHg. To manage raised ICP, patients were positioned at a 30° angle head up and received sedation, analgesia, neuromuscular blockade and osmotherapy with mannitol (0.5 g/kg) as required.

In the event of a rise in ICP in excess of 20 mmHg, refractory to the standard treatment (including surgical removal of haematoma), a barbiturate coma was induced.

Guidelines for the management of body temperature have been developed to form a clinical protocol. At our centre, temperature management is directed towards maintenance of normothermia; therapeutic hypothermia is not included as a part of routine neurocritical care. Briefly, the temperature management protocol involves a four level, step-up method for control of body temperature beginning with antipyretic drugs (level 1) with the addition of surface cooling (level 2) neuromuscular blockade (level 3) and intragastric cooling with ice cold water lavage (level 4) with an intention to achieve a target brain temperature of 37°C (normothermia). In step 2 of our body surface cooling protocol we applied wet (hand hot) cotton sheets to the patient's body (from chest to mid thigh) and renewed the sheets on an hourly basis when starting to dry. In step 4 of the protocol, patients received 500 ml of iced water into the stomach via a nasogastric tube and the residual volume was aspirated after 10 minutes. This procedure was repeated every 15 minutes for a maximum of five hours with regular clinical assessment of blood sugar and electrolytes during gastric lavage. Enteral feeding was resumed at the end of level 4 cooling.

Assessment of injury severity was made from the information obtained in the patient's case notes. Details of all the injuries sustained at the time of the accident were noted. The abbreviated injury scale (AIS) [12,13] was used to 'grade' the severity of trauma to the head. The AIS for the head region (Table 1) includes trauma to the brain and cranium. Patients were eligible for recruitment if brain temperature was being recorded during routine neurocritical care.

**Table 1:** Patient demographics: injury aetiology, brain pathology diagnosis and injury severity scores

Patient	Aetiology	Brain Pathology	Measurements made on (day after TBI)	Number of hours studied on ICU	^†^n	AIS	ISS
A	Fall	ICH	3	5	13	4	17
B	Fall	Bilateral frontal haematoma	5, 6	5	17	5	30*
C	Fall	SDH	3, 5	5	14	4	16
D	Fall	DAI	4	5	11	5	26
E	RTA	Temporal contusions	6, 7	5	20	4	16
F	RTA	DAI	2, 3	5	26	5	45*
G	Fall	ICH	2	5	12	5	25
H	Assault	SDH	5, 6	5	23	4	17
I	Assault	Temporal contusions	2	5	9	3	18*
J	Fall	SDH	2	5	10	4	16
K	RTA	SDH	3	5	11	4	16
L	Fall	SDH, SAH, cerebral contusions	4	5	21	5	42*
M	RTA	SDH, cerebral oedema	4	5	21	4	24*
N	RTA	Cerebral oedema, SAH, contusions	5	5	21	4	21*
O	Fall	Cerebral contusions	4	5	21	4	16
P	RTA	DAI	2	5	19	5	66*
Q	RTA	Cerebral oedema	4	5	21	3	17*
R	Fall	EDH, cerebral oedema, cerebral contusions	3	5	21	4	16
S	RTA	SDH, cerebral contusions	2	5	21	4	41*
T	RTA	SAH, cerebral contusions	2, 3	5	21	4	18*

* significant systemic trauma, ^†^number of measurements made. AIS 1 = minor injury; AIS 5 = the most severe of survivable injuries.

AIS = abbreviated injury scale; DAI = diffuse axonal injury; EDH = extradural haematoma; ICH = intracerebral haemorrhage; ICU = intensive care unit; ISS = injury severity score; RTA = road traffic accident; SAH = subarachnoid haemorrhage; SDH = subdural haematoma; TBI = traumatic brain injury.

### Temperature measurement

#### Intraparenchymal temperature

Brain temperature was measured continuously using a combined ICP/temperature probe (Neurovent-PTemp™, Raumedic AG, Münchberg, Germany). Although no published data are available to show the precision of the Neurovent-PTemp, recent unpublished data by the author indicates that sensor performance exceeds the manufacturer's stated accuracy.

The sensor was inserted into parenchyma, under aseptic conditions at the bedside or during emergency neurosurgery. Using aseptic techniques, the sensor tip was positioned 3 to 4 cm into deep white matter of the right frontal lobe, via a standard burr hole. Temperature measurements (alongside other routine vital signs and clinical parameters) were displayed in real-time via a patient data acquisition system (Marquette Electronics, Milwaukee, WI, USA), updated and stored to a bedside computer at 10-minute intervals.

#### Infra-red Thermometry

Infra-red techniques were used to obtain body temperature using either an established site and method (tympanic membrane thermometry) or a novel infra-red method (temporal artery thermometry). Both methods use a non-contact temperature measurement device to detect the infra-red energy emitted from a specific body site at temperatures above absolute zero (-273°C).

Measurement of tympanic membrane temperature was made using a Core-Check thermometer (Model 2090 IVAC Corporation, San Diego, California, USA). To ensure that the 'lens' was directed at the tympanum, the pinna was gently held and the thermometer inserted into the external auditory meatus, turned upwards and directed towards the eye. The probe remained briefly in this position until the machine 'bleeped' to signal a temperature reading.

To obtain a temporal artery temperature reading, a small, hand-held infra-red scanner (Model TAT 5000, Exergen, Watertown, MA, USA) was used. The temporal artery thermometer [10] incorporates an infra-red sensor which is placed on the skin at the centre of the patient's forehead. The thermometer housing includes a measurement button which, when pressed, allows measurement to begin. The infra-red sensor (at the head of the hand-held thermometer) is then swept horizontally along the forehead to the hairline (crossing part of the course of the temporal artery). Keeping the button pressed down, the sensor is then removed briefly from the skin and placed behind the earlobe to touch the skin overlying the mastoid process and in the manner recommended in the product guidelines. This skin tap is used to control for evaporative cooling of the forehead if the patient is sweating. If the temperature at the mastoid is greater than the measurement over the temporal artery then the temperature at the mastoid process will be recorded [11]. On release of the button, a digital temperature measurement is displayed. The infra-red thermometer provides an estimate of body core temperature using a proprietary algorithm which incorporates a factor compensating for measured ambient temperature [11]. In each patient a measurement of brain temperature, tympanic membrane temperature and temporal artery temperature was recorded as a temperature 'triplet'. Each measurement triplet was made every 15 to 30 minutes for a total duration of five hours.

### Statistics

To undertake this descriptive study, a target of 20 patients with severe TBI was set as a pragmatic sample size to test whether temporal artery temperature performs better to predict brain temperature after TBI than tympanic membrane temperature does. The Bland and Altman method [14] was used to display the spread of data points. Using standard meta-analysis calculations, the absolute difference between each brain and tympanic temperature pair and the corresponding brain and temporal artery pair was calculated.

## Results

**Figure 1:**
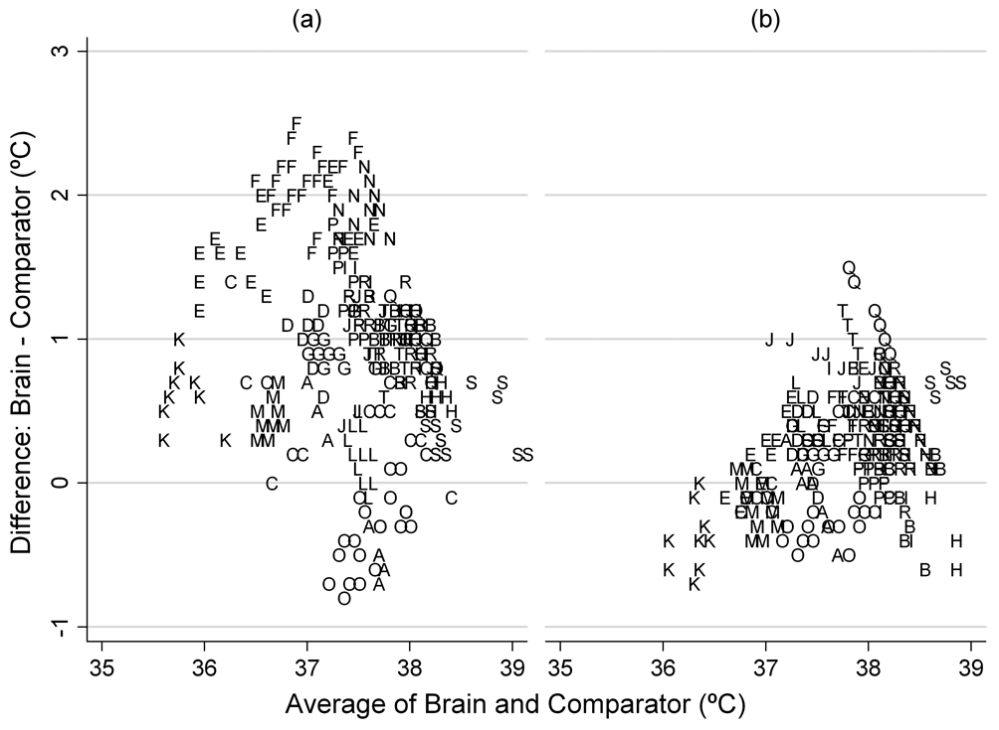
Bland and Altman plot graphs of the difference between brain temperature and its respective temperature pair (comparator) versus the average of the temperature pair. **(a) **Differences between brain and tympanic temperature readings. **(b) **Differences between brain and temporal artery readings (n = 353 data sets). The data points for each of 20 patients are distinguished by letters of the alphabet (A-T; Table 1).

Twenty patients (16 male, 4 female) aged 17 to 76 years (median 33 years) with severe TBI (median AIS 4) due to road traffic accidents (n = 9), falls (n = 9) or assault (n = 2) were each studied over the course of five hours during the first two to seven days (median three days) after injury. The ambient temperature of the ICU during the study ranged from 22.5 to 23.6°C (median 23.1°C). Between 9 to 26 (median 20) temperature measurement 'triplets' were obtained predominately during the period 10.00 to 15.00 hours on the day of the study. Three (15%) patients had diffuse axonal injury, 15 (75%) had haemorrhage and contusions, one patient (5%) had a bilateral frontal haematoma and one patient (5%) had cerebral oedema. Ten (50%) patients had significant systemic trauma in addition to severe brain damage (Injury Severity Score 17 to 66, median 27). Two sets of readings were provided by 353 temperature 'triplets': brain and tympanic membrane readings, and brain and temporal artery readings. The temperature measurements were made by one member of the research team (DK) only. Two patients (B and H) received surface cooling (level 2). Differences between brain and tympanic membrane temperature readings ranged from -0.8°C to 2.5°C and for brain and temporal artery temperature readings, -0.7°C to 1.5°C (Figure 1). The mean difference between brain temperature and tympanic membrane temperature was 0.91°C and the mean difference between brain temperature and temporal artery temperature was 0.26°C.

**Figure 2:**
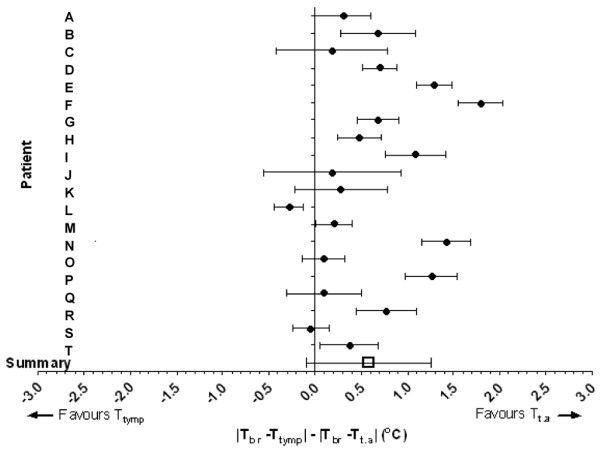
Weighted mean differences between brain and tympanic membrane temperatures and brain and temporal artery temperatures for each patient studied, with 95% confidence intervals. A positive value indicates that on average, temporal artery temperature is closer to brain temperature than tympanic membrane readings. |T_br _- T_tymp_| - |T_br _- T_t.a_| denotes the difference between absolute temperature differences of the respective brain-body temperature pairs. Data points to the right of the vertical line indicate that differences between brain and tympanic temperature readings are greater than for brain and temporal artery readings, i.e. the arrow to the right of the vertical line indicates that readings favour temporal artery temperature. The summary symbol (□) denotes the overall average by meta-analysis. T_br _= brain temperature; T_t.a _= temporal artery temperature; T_tymp _= tympanic membrane temperature.

**Figure 3:**
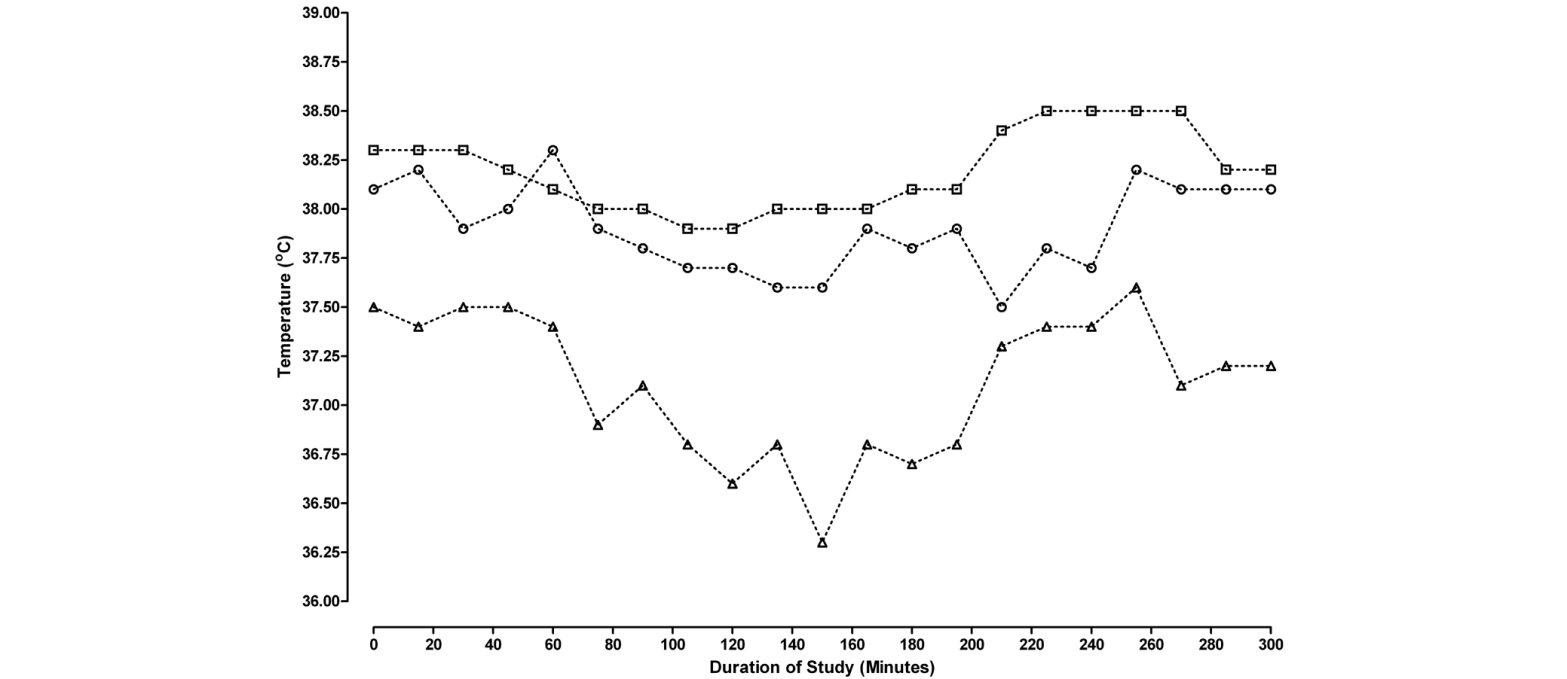
Temporal pattern of T_br _(boxes), T_t.a_. (O) and tympanic membrane temperature (△) for patient R during 270 minutes of study. T_br _= brain temperature; T_t.a _= temporal artery temperature.

The absolute temperature difference between brain temperature and tympanic membrane temperature pairs and brain temperature and temporal artery temperature pairs for each patient studied is given in Figure 2. This graph shows which of the two body temperatures agrees most closely with brain temperature. If both of the measurement techniques (temporal artery and tympanic membrane readings) were in agreement with brain temperature it would be reasonable to expect that there should be no difference between the temporal artery thermometer reading and the tympanic membrane reading to determine brain temperature; therefore, the mean difference between the two pairs of temperature differences should be zero. Using a standard meta-analysis method to describe these data, the mean weighted difference between the two infra-red measurement techniques (using absolute temperature values and ignoring the sign) is 0.58°C (Figure 2). This implies that overall, tympanic membrane temperature differs, in either direction, from brain temperature by an average of 0.6°C (95% confidence interval (CI) = 0.31 to 0.85, *P *< 0.0001) more than temporal artery temperature does. Figure 3 shows a typical example of the temporal pattern of brain, tympanic membrane and temporal artery temperature for one patient (patient R; Figure 2).

In this study, two patients were noted to be sweating on the forehead during the measurement period. In patient B, onset of sweating led to a 1°C increase in temporal artery temperature without a corresponding change in tympanic or brain temperature (or rectal temperature, data not shown). A similar, approximately 1°C rise in temporal artery temperature was noted once again (patient H) over a similar time period and again without corresponding effects on brain or tympanic (or rectal) temperature.

## Discussion

In this study we found that on average, for normothermic and febrile TBI patients, temporal artery temperature was closer to brain temperature than tympanic temperature was, by approximately 0.6°C. This suggests that within physiological and fever-range temperatures there is greater accuracy and less variability in the estimation of brain temperature using the temporal artery thermometer. As the patients in this study did not undergo therapeutic hypothermia, these results cannot be extrapolated to below-normal temperature readings.

Although studies in rodents clearly show that raised temperature leads to an increase in infarct volume after experimental cerebral ischaemia [15], there are no comparable data to confirm that raised temperature causes a worse outcome in patients with stroke or severe head injury. Even so, the current weight of opinion for brain injured patients is that a rise in body temperature (and by assumption, a rise in neuronal temperature) is damaging and should be treated [16]. There are, however, important gaps in our knowledge about the impact of raised (or below-normal) temperature on outcome in patients with brain damage. For example, it has not yet been established: if and how fever-range temperatures worsen outcome; whether control of fever improves outcome; or if hypothermia is appropriate for neurological patients. With regard to the role of fever-range temperatures on outcome after TBI, two recent studies from our centre [2] (and R.H. Sacho, unpublished MD thesis) suggest that a modest early fever of 39°C or below is not deleterious to outcome at either three or six months. If temperature does play a key role in influencing patient outcome in brain damaged patients, accurate measurement of 'at-risk' tissue (i.e. brain) must be a priority. However, it is not always possible to measure brain temperature directly. Furthermore, we must recognise a limitation to any clinical investigation involving brain temperature measurement because it is difficult to measure brain temperature directly at more than one site. We can not therefore be certain that measurements made in one focal area (e.g. uninjured tissue) represent the temperature in other brain regions (e.g. in areas of contusion, haemorrhage and ischaemia).

The search for a 'surrogate' non-invasive body site, which best reflects brain temperature remains of interest to clinicians. Tympanic membrane temperature is currently the most commonly used, non-invasive method of brain temperature estimation in the UK [7]; however, recent published data has raised concerns about its accuracy, the cause of which may be due to measurement error, user technique or 'true' temperature differences between the ears. Measurement of the temperature of the tympanum as a substitute for brain temperature has been justified because it is the closest anatomical structure to the brain that can be accessed without the need for surgery [11]. Most studies assessing the accuracy of tympanic membrane thermometry have been conducted in children. Craig and colleagues [10] in their systematic review found that the mean differences between body (rectal) and tympanic membrane measurements were small but the wide limits of agreement observed suggested that tympanic membrane temperature is not a good approximation of deep body core temperature. There is little information available, however, about the accuracy of the tympanic membrane temperature technique with regards to estimation of brain temperature.

Many studies have shown that blood flow in the head is altered in patients with severe TBI, most studies showing a tri-phasic blood flow pattern [17]. During the acute phase corresponding to the initial hours after injury, cerebral blood flow (CBF) is low, falling on average to approximately 50% of normal [17,18]. The second phase beginning around 12 hours after injury is marked by a rise in CBF that approaches or exceeds normal values in some patients, typically persisting for the next four to five days. A third phase of low CBF follows, lasting for up to two weeks [19-21]. As measurement of tympanic membrane temperature detects heat emitted (via the tympanum) from blood flowing through branches of the maxillary and middle meningeal arteries [11], temperature measurements in haemodynamically unstable patients may be different from that in healthy people or in patients who have a stable cardiovascular function.

One might propose that alterations in blood flow may also influence the temperature obtained using the temporal artery scanner but as the frontal branch of the superficial temporal artery lacks arteriovenous anastomoses, it is not subject to the same thermoregulatory vasomotor stimuli [22] as occurs in other skin regions. Thus the skin overlying the temporal artery may be an ideal site for temperature measurement, even under conditions of haemodynamic instability.

However, a note of caution should be considered in the estimation of brain temperature when sweating over the forehead is observed. We noted a 1°C rise in infra-red temporal artery readings during local (forehead) sweating. This finding might be explained by the fact that during the 'sweep' across the forehead (followed by the 'behind the ear' tap over the mastoid) the temporal artery thermometer records the 'peak' temperature value of the completed measurement. As the skin over the mastoid would be warmer than the skin over the cooler (sweating) forehead, this might offer an explanation for the apparently higher readings observed during forehead sweating. Wiping away visible sweat might improve the accuracy of the reading under such circumstances.

In a recent publication, tympanic membrane temperature was shown to drift from brain temperature by as much as 3°C [23]. In the present study we have shown that the average difference between brain and tympanic temperature readings was 0.9°C but individual readings could differ by up to 2.5°C. Such differences might be attributable to inaccuracies in measurement at either site, although as the brain temperature sensor was inserted directly into the brain parenchyma it is more likely to be inaccuracies using the tympanic membrane method. When using the infra-red tympanic membrane thermometer, the probe, once inserted into the ear, must 'see' the tympanic membrane [24]. If it does not, the infra-red radiation energy detected will be that of the ear canal rather than of the tympanum *per se*; the reading may therefore be inaccurate. Further inaccuracies can be avoided by ensuring the ear canal is free from cerumen [25]. This is clearly of clinical importance as tympanic temperature is currently one of the 'first-line' methods (along with skin folds, axilla and groin) for temperature measurement in UK neurocritical care patients [7]. How can we improve our ability to find a surrogate measurement when brain temperature monitoring stops or, as in many cases, is not performed at all? A possible solution is the infra-red temporal artery technique. A previous study [26] comparing temporal artery to pulmonary artery measurements in adults, however, showed poor performance. As far as we are able to tell, none have assessed the agreement between temporal artery and brain temperature.

## Conclusions

The infra-red temporal artery thermometer is a new option for clinicians to estimate brain temperature but there are a number of possible limitations to its use. For example, when sweat is observed over the forehead, the possibility for erroneous readings should be considered. In this study, differences between brain and systemic temperature methods were investigated in normothermic and pyrexial patients only. Whether comparable differences between brain and body sites occur in hypothermic patients (spontaneous and deliberate therapeutic hypothermia) require further investigation.

While this study is limited to a small sample size, on the basis of results presented, further work is needed to validate our findings in a larger population of brain injured patients where improvements in the conventional monitoring methods are desirable.

## Key messages

• In this pilot study temporal artery temperature performed better as a surrogate for brain temperature than tympanic temperature did.

• During visible sweating the performance of the temporal artery thermometer to reflect brain temperature may limit its usefulness as a brain temperature surrogate.

## Abbreviations

AIS: abbreviated injury scale; BTF: Brain Trauma Foundation; CBF: cerebral blood flow; CT: computed tomography; ED: emergency department; GCS: Glasgow coma scale; ICP: intracranial pressure; ICU: intensive care unit; TBI: traumatic brain injury.

## Competing interests

The authors declare that they have no competing interests.

## Authors' contributions

CC conceived and designed the study and wrote the paper. AV performed the statistical analysis. DK performed all temperature measurements and contributed to the manuscript preparation during an undergraduate medical student research project option. TR provided technical assistance and contributed to the preparation of the manuscript. All authors have given final approval of the version to be published.
